# Combination Drug Therapy for the Management of Chronic Neuropathic Pain

**DOI:** 10.3390/biom13121802

**Published:** 2023-12-16

**Authors:** Serena Boccella, Lidia De Filippis, Cristina Giorgio, Laura Brandolini, Meghan Jones, Rubina Novelli, Ezio Amorizzo, Matteo Luigi Giuseppe Leoni, Gaetano Terranova, Sabatino Maione, Livio Luongo, Manuela Leone, Marcello Allegretti, Enrico Maria Minnella, Andrea Aramini

**Affiliations:** 1 Research & Early Development (R&D), Dompé Farmaceutici S.p.A, Via De Amicis, 80131 Naples, Italy; serena.boccella@dompe.com (S.B.); cristina.giorgio@dompe.com (C.G.); 2 Research & Early Development (R&D), Dompé Farmaceutici S.p.A, Via S. Lucia, 20122 Milan, Italy; lidia.defilippis@dompe.com (L.D.F.); rubina.novelli@dompe.com (R.N.); manuela.leone@dompe.com (M.L.); enrico.minnella@dompe.com (E.M.M.); 3 Research & Early Development (R&D), Dompé Farmaceutici S.p.A, Via Campo di Pile, 67100 L’Aquila, Italy; laura.brandolini@dompe.com (L.B.); marcello.allegretti@dompe.com (M.A.); 4 Research & Early Development (R&D), Dompé US, 181 2nd Avenue, STE 600, San Mateo, CA 94401, USA; meghan.jones@dompe.com; 5 Pain Unit, San Paolo Hospital, 00053 Civitavecchia, Italy; amorizzoezio@gmail.com; 6 Pain Clinic Roma, 00191 Rome, Italy; 7 Azienda USL di Piacenza, 29121 Piacenza, Italy; matteolg.leoni@gmail.com; 8 Department of Medical and Surgical Sciences and Translational Medicine, Sapienza University, 00185 Rome, Italy; 9 Pain Unit, ASST Gaetano Pini, 20122 Milan, Italy; dr.gaetano.terranova@gmail.com; 10 Department of Experimental Medicine, University of Campania “Luigi Vanvitelli”, 80138 Naples, Italy; sabatino.maione@unicampania.it (S.M.); livio.luongo@gmail.com (L.L.)

**Keywords:** chronic pain, combination pharmacotherapy, co-crystal, analgesia

## Abstract

Chronic neuropathic pain (NP) is an increasingly prevalent disease and leading cause of disability which is challenging to treat. Several distinct classes of drugs are currently used for the treatment of chronic NP, but each drug targets only narrow components of the underlying pathophysiological mechanisms, bears limited efficacy, and comes with dose-limiting side effects. Multimodal therapies have been increasingly proposed as potential therapeutic approaches to target the multiple mechanisms underlying nociceptive transmission and modulation. However, while preclinical studies with combination therapies showed promise to improve efficacy over monotherapy, clinical trial data on their efficacy in specific populations are lacking and increased risk for adverse effects should be carefully considered. Drug-drug co-crystallization has emerged as an innovative pharmacological approach which can combine two or more different active pharmaceutical ingredients in a single crystal, optimizing pharmacokinetic and physicochemical characteristics of the native molecules, thus potentially capitalizing on the synergistic efficacy between classes of drugs while simplifying adherence and minimizing the risk of side effects by reducing the doses. In this work, we review the current pharmacological options for the treatment of chronic NP, focusing on combination therapies and their ongoing developing programs and highlighting the potential of co-crystals as novel approaches to chronic NP management.

## 1. Introduction

Pain is an unpleasant sensation, with a salient emotional component, that can be an adaptive and critical alert system, facilitating learned avoidance of potential tissue damage or other danger [1]. However, persistent, chronic pain becomes pathological and imposes substantial clinical, psychological, social, and financial burdens on society. Chronic pain is a critical global health concern, with chronic pain syndromes identified as one of the leading contributors to the global disease burden worldwide [2]. Neuropathic pain (NP), which arises from a disease or lesion of the somatosensory nervous system [1,3], represents a substantial proportion of chronic pain, with nearly one quarter of those with chronic pain and up to 10% of the global population experiencing chronic NP [4]. 

NP significantly impairs the quality of life and daily activities of patients, resulting in total health-care costs increasing to three times higher than in matched controls [5]. The underlying etiology of NP is multimodal and heterogenous, making its diagnosis and therapeutic management extremely challenging. Further complicating the picture, currently available analgesics often only convey partial pain relief for NP patients and are associated with dose-limiting side effects [6,7,8,9]. 

Effective approaches for the pharmacological management of NP will be those that leverage synergistic efficacy associated with multimodal therapies acting on converging mechanisms underlying NP in parallel. However, the wide-spread use of traditional combination therapy regimens is hindered by dose-limiting side effects and limited efficacy and safety data supporting the risk/benefit profiles for combinations proposed thus far. This narrative review describes the current treatment landscape for NP, including combination therapies and their ongoing development programs, and highlights co-crystallization as an innovative approach to drug combination which conveys several important advantages over combination therapy regimens. 

## 2. Pathophysiological Mechanisms of Chronic NP

Canonically, nociception is the activation of multimodal receptors, nociceptors, which transduce noxious stimuli into electrical signals and conduct from peripheral sensory nerves to the central nervous system (CNS) to drive the sensory experience of pain [10]. Types of pain are classified according to the pathophysiological mechanisms of nociceptor activation and duration. When pain reflects the mechanism and the severity of a sporadic and limited etiologic event, it can be defined as acute; but if it occurs regularly over a period of several months (i.e., more than 3 months), the pain process becomes chronic and pathological [3,11]. Nociceptive pain occurs when tissue damage (e.g., following internal or external injuries originating from accidents or medical procedures) activates nociceptors [10]. A newly defined category of pain, known as nociplastic pain, is characterized by the absence of obvious signs of sickness, injury, or somatosensory system lesion. In this case, pain arises from altered nociception and central sensitization, and the nervous system plays a central role [12]. Finally, pain arising from a somatosensory system lesion or disease is classified as NP [3].

The definition of NP has been elusive for the last 20 years. Its diagnosis and management remain difficult and to some extent arbitrary today. This is due in part to a lack of biomarkers and/or clinical tests, and the extremely dynamic nature of the nociceptive system. In 2019, the International Association for the Study of Pain (IASP) published a classification system to facilitate epidemiological investigations and health policy decisions surrounding the research and availability of multimodal pain management [3,11]. 

NP is usually chronic, defined as persistent or recurrent increased pain sensitivity or spontaneous pain stemming from a lesion or disease to the peripheral or central nervous system [1,3,11]. Somatosensory abnormalities in an area innervated by the damaged peripheral or central nerves are the primary complaint of patients with NP [11]. Symptoms affect both spontaneous and evoked pain perception and can include hyperesthesia, hypoesthesia, allodynia, and paresthesia. In addition to the presence of these symptoms and a pain syndrome, peripheral and/or central changes in one or more of the somatosensory systems, including non-nociceptive tactile and proprioceptive systems, thermoregulatory systems and visceral afferents, confirmed by clinical tests (e.g., electromyography, nerve or skin biopsies, CNS imaging, biochemistry and molecular biology), are required to receive a diagnosis of NP [1,13]. The etiology is multifactorial/multifaceted, as diseases or lesions throughout the entire pain perception path, from the peripheral nociceptor to the cortex, can drive NP syndromes. Peripheral NP can be caused by genetic mutations in key nociceptive receptors or ion channels or by a variety of axonal or demyelinating injuries [14]. Peripheral NP occurs due to acute events (e.g., trauma, amputation, and surgery), diseases (e.g., diabetes, and herpes zoster) infection/post-infection syndromes (e.g., Guillain-Barre syndrome), peripheral entrapment, spinal column degeneration, tumor infiltration, or adverse events associated with pharmacological or radiation treatments [14,15,16]. In contrast, central NP can be caused by lesions in the spinal cord, the spinothalamic tract, or the brain, with specific involvement of the brainstem, thalamus, and subcortical structures [1]. Common etiologies of central NP include post-stroke pain, multiple sclerosis, Parkinson’s disease, Alzheimer’s disease, amyotrophic lateral sclerosis, and syringomyelia. NP can also be classified as mixed NP when it is derived from a combination of peripheral and central somatosensory impairments, for example, as in lumbar or cervical radiculopathies [17,18,19]. 

NP is considered a distinct clinical entity, comprised of many syndromes which create a heterogenous group. Wide-ranging safe and effective therapies for NP patients across syndromes are an important unmet medical need. 

## 3. Pharmacological Treatment of Chronic NP: Drug Classes/Monotherapy 

Currently available pharmacological treatments used for the management of chronic NP are only able to mitigate the severity of symptoms rather than address the causative factors driving the disease [20]. According to the most recent evidence-based recommendations from the Neuropathic Pain Special Interest Group (NeuPSIG, Washington, DC, USA), first-line recommended treatments for NP include certain antidepressants and antiepileptic drugs, with tramadol and topical treatments recommended as first-line treatment if the pain is localized or as second-line treatment [7,20,21]. Third-line treatments include strong opioids or botulinum toxin type A-haemagglutinin complex (BoNTA). Notably, there was no conclusive evidence from the meta-analysis conducted by the NeuPSIG for the efficacy of cannabinoids, tapentadol, sodium channel-targeting antiepileptics, selective serotonin reuptake inhibitors or other topical drugs [7]. Further, NeuPSIG analyses reveal only modest outcomes in positive trials for NP across drug classes. Based on a meta-analysis of published and unpublished trials, even the first-line recommended treatments were associated with only moderate efficacy and low to moderate tolerability for most agents [7,20,21]. 

First-line antidepressants recommended for the treatment of peripheral or central NP include tricyclic antidepressants (TCA) (amitriptyline and nortriptyline) and serotonin-noradrenaline reuptake inhibitors (SNRI) (duloxetine and venlafaxine) [7,20,21]. Despite a strong GRADE (Grading of Recommendations Assessment, Development and Evaluation) recommendation (SNRIs, high-quality evidence; TCAs, moderate-quality evidence), analyses indicated only moderate effect sizes or low to moderate tolerability [7]. TCAs and SNRIs were initially developed for the treatment of mood disorders. The mechanisms through which they exert analgesia are still under investigation, but are thought to stem from descending noradrenergic signaling and/or N-methyl-D-aspartate receptor and ion channel blockade [22]. Despite some successful clinical trials, TCAs are estimated to provide pain relief in only 1 of every 2 to 3 patients with peripheral NP and SNRIs in 1 of every 4 to 5 [22]. 

Evidence supporting the use of the first-line recommended antiepileptic drugs, gabapentinoids, or GABA-mimetic antiepileptic drugs [23], is the strongest of those assessed by the NeuPSIG and applicable to both central and peripheral NP (strong GRADE recommendation, high-quality evidence, moderate effect size, moderate to high tolerability) [7,20]. Gabapentinoids, such as gabapentin and pregabalin, are first- and second-generation α2δ inhibitor ligands, respectively, and both are approved for use as adjunctive therapy in pain control. Their analgesic efficacy is thought to stem from the inhibition of injury-induced spinal neuronal excitability via the α2δ subunit of pre-synaptic calcium channels in the spinal cord. They can also act at the supraspinal level by modulating the dopamine-dependent negative affective states that have been reported in pain [24] or modulating the release of substance P-induced neurotransmitter release [25]. Indeed, gabapentin has been shown to be effective in the treatment of post-herpetic neuralgia, diabetic neuropathy, trigeminal neuralgia and pain syndromes following spinal cord injury, and also for deep tissue pain and hyperalgesia [26]. Interestingly, while showing no effect on acute cutaneous nociception induced by a brief thermal stimulus, it was effective in reducing temporal summation of skin stimuli at pain threshold intensities, suggesting gabapentin as a reference drug for the treatment of neurogenic pain [27]. Gabapentin was approved for use as an adjunct treatment for partial epileptic seizures in adults and children in 1993, and then for the treatment of chronic pain, in particular NP syndromes [28,29]. 

Tramadol (an opioid with additional monoaminergic activity [30,31]) and topical treatments (if the pain is localized), such as capsaicin high-concentration patches and lidocaine, may be used as a first-line or second-line treatments but are associated with weaker effect sizes [7,21]. Specifically, tramadol is recommended for central or peripheral NP and is associated with a weak GRADE recommendation and moderate-quality evidence, while capsaicin patches and lidocaine are recommended for peripheral NP and associated with weak GRADE recommendations based on either high-quality or low-quality evidence, respectively [7]. Strong opioids are recommended as third-line treatments for both peripheral and central NP, but this is a weak GRADE recommendation based on only moderate-quality evidence and recent increases in prescription opioid-related overdose deaths emphasize the gravity of the abuse liability associated with opioid use [7,32,33]. The development of tolerance to opioids, counteracting the effects of repeated administrations, also limits their long-term use in patients with NP. Lastly, Botulinum toxin type A-haemagglutinin complex (BoNTA) may help some NP patients, but is only recommended for peripheral NP (weak GRADE recommendation, low-quality evidence) [7]. 

Nonsteroidal anti-inflammatory drugs (NSAIDS), or nonselective cyclo-oxygenase-1/2 (COX1/2) inhibitors, as well as selective COX2 inhibitors, are also commonly used to manage inflammation and pain in NP, particularly in sub-acute and acute-on-chronic phases [34,35]. NSAIDs exert efficacy both by controlling peripheral inflammation and through either the disinhibition of endogenous opioid signaling or activation of endocannabinoid signaling at the level of the spinal cord, periaqueductal gray (PAG) and rostral ventromedial region of the medulla [36,37,38,39,40]. However, tolerability of NSAIDs and selective COX2 inhibitors is limited by their risk for renal toxicity, gastrointestinal toxicity, cardiotoxicity, and nephrotoxicity [41,42,43,44], discouraging their use in patients with pre-existing pathologies [45,46,47,48,49,50]. NSAID-associated gastropathies have been reported to cause 2600 deaths and 20,000 hospitalizations per year in rheumatoid arthritis patients alone [51]. While selective COX2 inhibitors spare the gastrointestinal toxicity of COX1, improving gastric tolerability, they are associated with considerable cardiovascular risk [41]. 

While these recommendations highlight pharmacological agents with potential efficacy in some NP patients, it is well known that the benefits of single-drug treatments are limited due to incomplete efficacy and dose-restricting adverse effects [6,7,8,9]. High risks of dose-related adverse reactions prevent the use of doses sufficient to produce complete pain relief [7]. Complicating the picture, risks for adverse effects may also differ depending on the specific etiologies of NP and need to be considered on an individual patient level [7]. The lack of clear efficacy of monotherapies in NP can be explained by the converging mechanisms and multiple potential pathologies known to contribute to chronic NP [52]. For this reason, combination therapy regimens that take a multimodal approach to treatment of chronic NP have also been evaluated [53]. 

## 4. Pharmacological Treatment of Chronic NP: Combination Therapy 

### 4.1. General Considerations

A multimodal approach to chronic NP management allows the modulation of multiple transmission pathways and enables individual pharmacological agents to act with potentially additive or synergistic effects (i.e., improvements in efficacy greater than the theoretical summation of the two analgesic effects). Drugs with distinct pharmacological mechanisms of action acting at different sites of nociceptive signaling will optimize efficacy [54,55]. Ideally, two distinct classes of drugs used in combination will have non-overlapping side effect profiles and minimal potential for either adverse interactions with other commonly used drugs or for the exacerbation of existing patient comorbidities [56,57]. When synergistic improvements in efficacy also render smaller doses of a given agent sufficient to achieve pain relief, combination therapy could also lead to improved tolerability [58,59,60,61]. 

Collectively, approximately half of patients with NP are reported to receive at least two analgesic drugs for pain management [62,63,64]. Different combinations of NSAIDs, gabapentinoids, opioids, and antidepressants have been investigated so far and have shown some efficacy for the management of NP across syndromes [65,66,67], and specifically for postoperative pain [68,69,70,71], rheumatological conditions (e.g., osteoarthritis or fibromyalgia) [72,73,74,75,76], diabetic neuropathy [77,78], back pain [79], post-herpetic neuralgia [80], and cancer-related pain or chemotherapy-induced peripheral neuropathy [81,82]. However, preclinical and clinical data describing the safety and efficacy of combination therapies are inconsistent [83,84]. Fine-tuning the balance between positive and adverse drug-related effects in all pain conditions, as well as consideration of individual patient characteristics (i.e., response to previous analgesics, the duration and severity of symptoms, and the presence of comorbidities) and the pathogenic mechanisms driving the NP are crucial for the success of combination therapies. Preclinical studies in which animal responses to noxious stimuli (e.g., heat, electrical current, or mechanical stress) or inflammatory pain (e.g., peripheral injections of carrageenan, formalin, complete Freund’s adjuvant, or monoiodoacetate) engage distinct underlying pathophysiological mechanisms are useful to gauge the preclinical efficacy of candidate combination therapies in this context [85,86,87,88,89]. Ongoing programs for combination therapy regimens for NP management aim to identify drug combinations with both improved analgesic efficacy and improved tolerability/reduced risk for side effects. Preclinical and clinical trial data on the efficacy and safety of specific combination therapy regimens evaluated for chronic NP management are summarized below and in Table 1. 

### 4.2. Preclinical and Clinical Data on Combination Therapy 

#### 4.2.1. NSAIDs and Opioids (Including Tapentadol/Tramadol)

NSAIDs are widely used in the treatment of various inflammatory pain states, including some NP syndromes. Because of their well-established, dose-limiting gastric and cardiovascular tolerability issues, they are often combined with analgesics of several classes that act through different mechanisms. The combination of dexketoprofen and tramadol has been identified as a valuable combination with synergistic improvements in analgesic efficacy. Tramadol acts as a weak μ-opioid receptor agonist and as a serotonin/noradrenaline reuptake inhibitor [30,31]. This combination leverages analgesic efficacy of relatively weak opioid activity in the spinal cord, enhanced descending noradrenergic contributions to pain inhibition, tramadol-driven modulation of microglia, and the anti-inflammatory activity characteristic of NSAIDs [72,90]. The combination of dexketoprofen and tramadol was associated with synergistic efficacy in preclinical models of nociceptive/postoperative (plantar incision) pain (i.e., tail flick, acetic acid writhing test, von Frey) [90,94] and inflammatory/chronic osteoarticular pain [95]. However, analyses of gastrointestinal transit following this combination revealed an increased risk for constipation, with dexketoprofen reversing the effects of tramadol in this context [94]. In another preclinical model of NP, the spared nerve injury (SNI) model, there is also preclinical evidence to support synergistic analgesic efficacy of ketorolac and tramadol for peripheral neuropathic pain [96]. 

Clinical trials revealed the synergistic efficacy of dexketoprofen and tramadol for acute postoperative pain management, with an improved longevity of the analgesic efficacy and comparable, but not improved, safety profiles relative to those of each drug administered as monotherapy [91,92,93]. Ketorolac has been shown to reduce acute postoperative pain and opioid use in both pediatric and adult populations [68,69,70,71], suggesting the combination of low-dose opioids with ketorolac as an improved therapeutic approach over monotherapies. However, two studies suggested that these benefits are not maintained beyond the acute postoperative phase [68,71]. A meta-analysis of 13 studies of the effect of perioperative ketorolac on postoperative analgesia outcomes demonstrated that only the higher dose of ketorolac, 60 mg, was associated with a reduction in postoperative opioid requirements, whereas 30 mg ketorolac was not sufficient [71]. 

#### 4.2.2. NSAIDs and Gabapentinoids/Antiepileptics

Several studies have evaluated analgesia following combinations of NSAIDs with antiepileptics, including gabapentin and pregabalin. With this combination, in addition to peripheral NSAID anti-inflammatory activity counteracting central sensitization and pain maintenance, spinal neurotransmitter spill over is mitigated by the gabapentinoid-induced inhibition of the α2δ subunit of presynaptic calcium channels. A known mechanism through which gabapentinoids and NSAIDs may interact is through the inhibition of the membrane translocation of protein kinase C epsilon type (PKC𝜀) in sensory neurons. This is an inflammatory-dependent process underlying pain maintenance [130], and, notably, paracetamol has been shown to potentiate the gabapentin-mediated blockade of PKC𝜀 membrane translocation induced by the pronociceptive peptides, bradykinin and prokineticin 2 in vitro [131]. 

Synergistic efficacy was observed in preclinical models of localized inflammatory pain (i.e., formalin- or carrageenan-induced) with several gabapentinoid/NSAID combinations including gabapentin–naproxen [97], pregabalin–naproxen [97], gabapentin–ibuprofen [98], and oxcarbazepine–ibuprofen [99]. The synergistic boost in efficacy from coadministration of ibuprofen with oxcarbazepine resulted in a reduction in the effective dose required to produce 50% analgesic efficacy (ED_50_) from a theoretical additive ED_50_ of 67.63 ± 5.44 mg/kg to an experimentally observed ED_50_ of 35.71 ± 2.90 mg/kg. A reduction in the dose required to produce sufficient analgesia with these combinations could substantially reduce the risk of adverse effects for patients with NP [99], but clinical studies are needed to investigate whether this synergistic efficacy translates clinically. 

Combination therapy with gabapentin–diclofenac exhibited synergistic efficacy in both postoperative and inflammatory pain models. Intrathecal coadministration of gabapentin and diclofenac at low doses of each agent insufficient to produce anti-hyperalgesia when administered alone (gabapentin, 4 µg; diclofenac, 2 µg) significantly reduced mechanical hyperalgesia when administered in combination in a preclinical model of postoperative pain (hindpaw incision) [100]. Similarly, synergistic efficacy was observed with this combination in inflammatory pain (i.e., formalin-induced), with an improvement in the ED_30_ value for diclofenac–gabapentin (administered directly into the hind paw) from the theoretical additive value of 597.5 ± 87.5 µg to the experimentally observed value of 170.9 ± 26.07 µg [101]. 

Synergistic efficacy greater than the theoretical sum of the effects produced by each drug alone was also observed in rat models of chronic constriction injury (CCI) and SNI with combinations of NSAIDs and gabapentinoids. In a rat model of CCI, synergistic anti-allodynic and anti-hyperalgesic effects were observed following combination therapy with meloxicam (1.0 mg/kg) and gabapentin (10 mg/kg) [102]. Interestingly, this was the only dose combination to produce synergistic improvements, while the combinations of meloxicam administered at doses of 0.1, 0.31, 3.2, or 10 mg/kg with 10 mg/kg gabapentin produced only an additive benefit. Finally, ketorolac and pregabalin also conveyed a synergistic benefit in an SNI model of peripheral mononeuropathy [96]. In this model, the effect of ketorolac and pregabalin on mechanical allodynia was notably stronger than that of ketorolac and tramadol. 

Data in humans for NSAID/gabapentinoid combinations are limited. However, combination therapy with pregabalin and celecoxib for the treatment of chronic low-back pain was reported to be superior to the respective monotherapies [79]. 

#### 4.2.3. Gabapentinoids and Opioids (Including Tapentadol/Tramadol)

In a preclinical study examining the effects of various combinations of pregabalin with duloxetine, venlafaxine, tramadol, and celecoxib on mechanical allodynia in rats with an L5 spinal nerve ligation, pregabalin with tramadol was the only combination to show synergistic anti-allodynic effects [103]. In the same study, pregabalin and duloxetine exhibited additive, but not synergistic efficacy, while pregabalin and venlafaxine appeared to be antagonistic. Of note, although combination treatment in the management of chemotherapy-induced peripheral neuropathy (CIPN) is still controversial, combination therapy with pregabalin and tramadol was found to have positive outcomes in taxane-induced peripheral neuropathy [82].

There is preclinical and clinical evidence supporting a synergistic effect of the combination of gabapentin and stronger opioids as well [104,105]. Synergistic anti-allodynic and anti-hyperalgesic effects of combination therapy with gabapentin and morphine greater than either agent administered as monotherapy or the theoretical additive effect of the combination were observed following CCI with the von Frey and acetone tests in an NP model in rats [105]. Gabapentinoid–opioid combinations have been evaluated also in several clinical studies in various NP patient populations; however, efficacy results have been inconsistent and safety and tolerability concerns remain about the risks for side effects with low-dose pregabalin and opioids [65,66]. A randomized open-label controlled trial of the addition of gabapentin to opioid use (oral tramadol, transdermal fentanyl, or sustained-release morphine capsules (pooled)) for the treatment of NP associated with cancer demonstrated a clear benefit of combination therapy over opioid therapy alone [104]. This is consistent with another study showing that combination therapy with gabapentin and opioids was effective in reducing the total pain score in cancer-related NP refractory to standard opioid regimens without severe side effects [81]. However, it is important to note that the risk for respiratory depression has to be carefully considered in geriatric populations. 

Despite the observed efficacy of several single drugs in diabetic peripheral neuropathic pain (DPNP), only approximately half of patients respond to treatment, with many reporting residual symptoms [78,132]. One randomized, double-blind, placebo-controlled trial demonstrated a significant improvement in pain relief with the addition of oxycodone to gabapentin treatment, relative to gabapentin treatment alone, in patients with DPNP [78]. 

Importantly, while the addition of gabapentin to an existing opioid regimen for the treatment of severe pain may lower the opioid dose required for pain relief, it brings with it additional risk for adverse effects associated with gabapentin. A 2022 meta-analysis of the risk of adverse events associated with opioid therapy vs. combination opioid and pregabalin therapy revealed that combination therapy may increase the risk for CNS depression and mortality, despite a tolerable gastrointestinal profile [66]. Thus, while several clinical trials suggest improvements and/or reductions in required opioid doses over monotherapy with gabapentinoid-opioid combination therapy for various syndromes of NP, safety remains an important concern with the doses required.

The combination of morphine with pregabalin markedly reduced pain scores relative to either agent as monotherapy in patients with several forms of NP, including back surgery syndrome, post-herpetic neuralgia, radiculopathy and stenosis of the spinal medullary canal [106]. This effect was achieved with lower mean dosages of morphine and pregabalin relative to doses required for monotherapy with each agent. Consistent with this, in a multicenter trial including patients with NP syndromes (i.e., back surgery syndrome, stenosis of the medullary spinal canal, post-herpetic neuralgia, and painful diabetic neuropathy), the combination of pregabalin and oxycodone was superior to pregabalin alone and caused fewer adverse effects than either oxycodone alone or pregabalin alone, allowing a dose reduction of both medications compared to the respective effective doses required for monotherapy [67]. However, a recent trial which included patients with post-herpetic neuralgia or painful diabetic neuropathy treated with pregabalin and oxycodone failed to demonstrate any beneficial effects of combination therapy compared to placebo or monotherapy [84]. Notably, differences in mechanisms driving neuropathic pain may complicate the interpretation of the results of studies which pool NP patient populations. 

#### 4.2.4. Gabapentinoids and Antidepressants

Combination therapy with gabapentinoids and antidepressants has also been examined in the context of DPNP, with inconclusive results. Patients with diabetic neuropathy treated with gabapentin in combination with venlafaxine reported pain relief and improvements in quality of life compared to those in a placebo group and to those who received gabapentin and placebo [77]. Similarly, in a double-blind randomized controlled crossover trial, the combination of nortriptyline with gabapentin in patients with diabetic polyneuropathy or postherpetic neuralgia conveyed better efficacy as compared to the single molecules given alone [107]. OPTION-DM, a multicenter, randomized double-blind crossover trial in patients with DPNP, evaluated three combination treatment arms: amitriptyline supplemented with pregabalin, pregabalin supplemented with amitriptyline, and duloxetine supplemented with pregabalin, with each arm lasting 16 weeks. In this trial, all three combination therapies exerted an analgesic effect and combination therapy led to a stronger improvement in daily pain than did monotherapy [108]. In contrast, in the COMBO-DN trial, low-dose duloxetine and pregabalin combination therapy was considered to be effective and safe, but not superior to high-dose monotherapy of either agent alone [109].

In a clinical study conducted by Chakrabarty and colleagues, the efficacy of pregabalin (150 mg) monotherapy, amitriptyline (25 mg) monotherapy, and low-dose pregabalin (75 mg) plus amitriptyline (10 mg) as combination therapy for reducing NP symptoms was investigated [110]. NP symptom inventory score (NPSI) significantly decreased in all groups, but combination therapy with lower doses yielded a better tolerability profile than did the monotherapy regimens. 

#### 4.2.5. Additional Combination Therapies 

In preclinical studies, combination therapy with metformin, a widely used first-line antihyperglycemic agent for type 2 diabetes, and ibuprofen, aspirin, tramadol or pregabalin led to a significantly better reduction of carrageenan-induced hyperalgesia as compared to monotherapy of each compound [111]. Similar synergistic analgesia was observed with diclofenac coadministered with the H1 antihistaminic pyrilamine in a rat model of carrageenan-induced paw edema [112]. In this study, in addition to synergistic improvements in anti-inflammatory and anti-nociceptive efficacy, diclofenac and pyrilamine combination therapy reduced gastric liability. Interestingly, natural substances can boost the anti-inflammatory effects of NSAIDs. The NSAID diclofenac exhibited improved effects on inflammation and nociception (i.e., formalin- or carrageenan-induced) and gastric injury in rats when combined with Matricaria chamomilla extract [113], the naturally occurring unsaturated monocyclic sesquiterpene alcohol–bisabolol [114], or docosahexaenoic acid (DHA), a long-chain polyunsaturated fatty acid (PUFA) [115]. In the CCI rat model of NP, meloxicam and refocoxib showed an improvement in anti-hyperalgesia when administered in combination with aminoguanidine hydrochloride, an inducible nitric oxide synthase (iNOS) inhibitor, relative to the respective monotherapies [116]. 

Gabapentin-metamizol [117], and oxcarbazepine–paracetamol combinations also exhibited anti-nociceptive improvements in models of inflammatory pain (i.e., formalin- or carrageenan-induced) [99,118]. In a partial spinal nerve injury model, combination therapy with subeffective doses of gabapentin and pregabalin potentiated the therapeutic efficacy of spinal cord stimulation, a tool used for neuropathic pain relief, as evaluated via the von Frey test [119]. Extracellular recordings confirmed that this low-dose combination enhanced the suppression of dorsal horn neuron hyperexcitability. 

Cannabinoids have been proposed as useful analgesics with weaker central psychological effects than opioids and have been evaluated in combination therapy regimens [133]. Indeed, one study demonstrated that intra-plantar coadministration of anandamide and ibuprofen or anandamide and rofecoxib ameliorated mechanical allodynia and thermal hyperalgesia in a model of partial sciatic nerve ligation, and suggested local use of cannabinoids as a potential way to take advantage of their anti-nociceptive nature and limit unwanted central effects [120]. In another study, systemic administration of WIN 55,212-2, a potent non-selective cannabinoid receptor agonist, and ketorolac produced an additive analgesic effect in the acetic acid-induced writhing test (inflammatory visceral pain), but no effect or added benefit of ketorolac in addition to WIN 55,212-2 in the thermal tail flick test was observed [122]. Combination therapy with WIN 55,212-2 and ibuprofen also exhibited synergistic efficacy in the formalin-induced model of inflammatory pain in rats [123]. 

Preclinical evidence also supports further evaluation of the potential for combination therapy with cannabinoids and opioids in the treatment of NP [133]. Combination therapy with morphine and WIN55,212-2 in rats exposed to chronic constriction injury [124] and combination therapy with tramadol and PhAR-DBH-Me, a synthetic cannabinergic compound, in rats exposed to spinal nerve ligation [125] both led to synergistic analgesic efficacy. Similarly, JWH015, a cannabinoid 2 receptor agonist, and morphine treatment led to synergistic analgesic efficacy in models of post-operative pain (plantar incision) and spared nerve injury [126]. In this study, the addition of JWH015 reduced the gastrointestinal side effects and conditioned place preference associated with morphine treatment [126]. In contrast, additive, but not synergistic, analgesic effects were observed with combination treatment with cannabidiol and tramadol in the streptozotocin-induced rat model of diabetic neuropathy [127] and with PhAR-DBH-Me and tramadol in a model of cisplatin-induced neuropathy [125]. Ultimately, despite preclinical evidence of the anti-nociceptive effects of cannabinoid receptor 1/2 (CB1/2) agonism and fatty acid amide hydrolase (FAAH) inhibition, translation of these preclinical findings into clinical data has been limited.

The coadministration of ibuprofen and paracetamol in fixed-dose combinations (FDC) has been suggested for pain management and has demonstrated synergistic efficacy in several contexts. It has been recommended as a preferred option relative to either agent as monotherapy for the treatment of headaches, odontalgia, earache, musculoskeletal pain, and postoperative pain in pediatric patients [134]. This combination has also been evaluated for pain management in adult patients. While a meta-analysis of nine clinical trials revealed a significant improvement in outcomes of patients treated with FDC ibuprofen and paracetamol relative to placebo, its value relative to either administered as monotherapy was not evaluated and safety analyses were inconclusive [135]. 

Reports of additional potential combination therapies in the context of central NP secondary to either stroke or spinal cord injury have also been reported. A case study of a patient who presented with post-stroke pain with multiple comorbidities reported improvements in neuropathic pain symptoms following combination therapy with prednisone and low-dose gabapentin [136]. In patients with NP after a spinal cord injury, synergistic analgesic efficacy was observed with morphine-and-clonidine combination therapy [128], though most patients required rescue treatment during the course of this trial, and with ketamine as an add-on to gabapentin [129]. 

### 4.3. Unmet Medical Needs 

Collectively, preclinical and clinical studies have provided a proof of concept and shown promise for some multimodal combination therapy regimens to convey additive or synergistic improvements in efficacy for NP. This represents an important achievement in the field of NP management warranting further investigation. However, there are several obstacles remaining before any specific combination therapy can be widely used for NP. The risk of side effects is still a substantial limiting factor, with a lack of clear safety and efficacy data and substantial risk for adverse events in specific NP populations. Further to this, combination therapy regimens may be more difficult for patients and caregivers to adhere to, especially in pediatric populations [137]. We argue that co-crystallization has the potential to capitalize on the synergistic efficacy between classes of drugs while also simplifying adherence and minimizing the risk of side effects by reducing the doses, potentially addressing many of these remaining challenges in the poorly managed NP treatment landscape. 

## 5. Pharmacological Treatment of Chronic NP: Co-Crystallization

**Figure 1:**
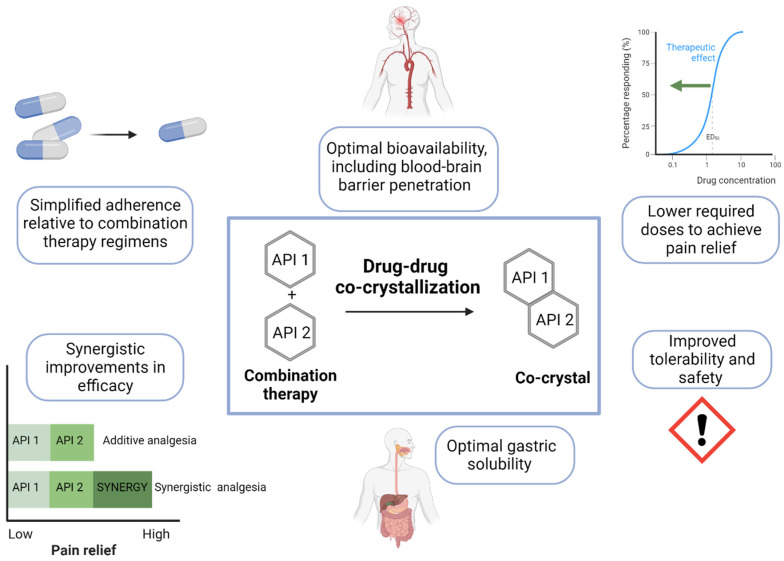
Potential advantages of co-crystals in the treatment of chronic NP. Drug-drug co-crystals can convey distinct advantages over combination therapy regimens including simplified adherence, optimal bioavailability and blood–brain barrier penetration, lower doses, improved tolerability, improved solubility, and synergistic improvements in analgesic efficacy. API, active pharmaceutical ingredient.

Co-crystallization has emerged in pharmaceutical science not only as solution for poorly soluble drugs, but also as an innovative pharmacological approach to drug combination which allows for the optimization of physicochemical and pharmacokinetic properties. In drug-drug co-crystallization, the aggregation of two or more active pharmaceutical ingredients (APIs) via noncovalent interactions yields a new single crystal with unique physicochemical properties but the same intrinsic chemical identity and pharmacological activity of the native molecules [138,139]. Thus, potentially, drug–drug co-crystals can leverage the synergistic efficacy of distinct classes of drugs while simultaneously optimizing bioavailability and minimizing the required doses and associated side effects, providing significant advantages over combination therapy regimens (Figure 1). 

Novel co-crystals formed from two analgesics have been synthesized. Two recent reports of novel co-crystals have demonstrated improved physicochemical properties that would be expected to convey benefits in vivo once evaluated. A co-crystal system containing febuxostat and piroxicam, both approved for the treatment of gout, exhibited improved solubility of both febuxostat and piroxicam and an improved dissolution rate of piroxicam [140]. In another example, co-crystallization of meloxicam with aspirin led to significantly improved meloxicam kinetic solubility, with improved oral bioavailability and higher maximum plasma concentration, which could substantially improve the time required for the drug to reach therapeutic concentrations [141]. Finally, drug–drug co-crystals of duloxetine and naproxen [142], diclofenac acid and ethyl diclofenac [143] or anti-inflammatory agents like ibuprofen, naproxen, ketoprofen, and flurbiprofen with levetiracetam [144] have also been reported. 

Two novel co-crystals have been evaluated in vivo for pain management (Table 2). Almansa and colleagues were the first to report the development of co-crystals containing rac-tramadol hydrochloride and celecoxib and demonstrate synergistic efficacy in vivo [145,146]. As described previously in this review, tramadol and celecoxib represent distinct drug classes with complementary mechanisms targeting multiple central and peripheral NP pathways, with celecoxib being suggested to have improved cardiovascular safety relative to other NSAIDs [147,148]. The co-crystal, called Seglentis^®^ has been evaluated for the treatment of acute postoperative pain. In this context, it exhibited an improved pharmacokinetic profile, with a faster intrinsic dissolution rate of celecoxib relative to celecoxib alone and a slower intrinsic dissolution rate of tramadol relative to tramadol alone [145,146]. The lower peak concentration of tramadol and faster analgesic onset of celecoxib in the co-crystal is an advantage over combination therapy of the two agents as it could convey a substantially improved risk/benefit ratio. Consistently, the co-crystal was more effective than the single drugs, and more effective than the theoretically calculated additive benefit of the two drugs, in reducing acute postoperative pain without potentiating the risk for adverse effects [145,146,149]. Specifically, the co-crystal was superior to either tramadol or celecoxib alone in exerting synergistic mechanical allodynic and thermal analgesic effects in the postoperative pain model, with experimental and theoretical additive ED_50_ values of 2.0 ± 0.5 mg/kg and 3.8 ± 0.4 mg/kg for mechanical allodynia and 2.3 ± 0.5 gm/kg and 9.8 ± 0.8 mg/kg for thermal hyperalgesia, respectively [149]. Importantly, the synergistic anti-nociceptive effects were comparable to strong opioids (morphine 2.5 mg/kg and oxycodone 5.8 mg/kg) but with improved tolerability. 

Phase I clinical studies confirmed that the co-crystal was associated with the expected improved pharmacokinetic profile relative to either of the individual formulations or their combination [150]. Subsequently, Phase II and III studies confirmed the significant improvement in the risk/benefit ratio observed with the co-crystal relative to either agent alone [151,152,153]. Seglentis^®^ is now approved for the treatment of acute pain in adults that is severe enough to require an opioid analgesic for which other treatments are inadequate [154]. 

Building on studies that extensively described the distinct pharmacodynamic profiles of ketoprofen lysine (KLS) and gabapentin, which act on peripheral inflammation and spinal plastic changes at the dorsal horn and descending levels underlying neuropathic pain, respectively, we have recently developed a new ternary drug–drug co-crystal containing ketoprofen, lysine and gabapentin (KLS-GABA) in a 1:1:1 stoichiometric ratio. 

Early in vitro studies evaluating the pharmacological combination of KLS and gabapentin (i.e., single agents in a mixture) provided initial proof-of-concept evidence that coadministration with KLS can improve gabapentin’s analgesic effects by enhancing its inhibition of PKC𝜀 translocation on the membrane of sensory neurons [130,155]. In parallel, gabapentin stimulated gastric mucosa repair and protection pathways, with substantially reduced stress-induced proinflammatory signals, including nuclear factor kappa light chain enhancer of activated B cells (NFκB) nuclear expression, observed in gastric mucosa [155], thus suggesting that the combination with gabapentin has the potential to improve the gastrointestinal tolerability of KLS [155]. The KLS-GABA co-crystal was also associated with a significantly increased gastric solubility of ketoprofen, which showed a supersaturation profile and a reduced particle aggregation in solution compared to KLS and gabapentin alone or in combination, resulting in a higher systemic exposure of ketoprofen in vivo. The supersaturation effect observed with the KLS-GABA co-crystal can substantially improve performance in vivo over combination therapy [156], as it optimizes the bioavailability of ketoprofen, which is known to have low solubility, not only by increasing its solubility level at equilibrium but also maintaining that solubility level for relatively long periods with a low rate of phase transformation [139,157]. Consistent with this, blood-brain barrier (BBB) permeation studies showed an increased central permeation of KLS and gabapentin when administered in combination versus the single drugs, with the co-crystal displaying a higher BBB influx/efflux rate compared to the mixture [155].

In vivo, KLS-GABA co-crystal was strikingly more efficient in treating both inflammatory and neuropathic pain and reducing spinal neuroinflammation than either agent administered as monotherapy or combination therapy [155]. Further to this, the protective effect of gabapentin on the gastrointestinal tolerability of ketoprofen was stronger with the co-crystal than with the combination therapy, with a stronger inhibition of NFκB and interleukin-8 (IL-8) observed with the co-crystal than with the combination. The co-crystal was also associated with an increase in gastric COX1 expression, which plays a crucial role in the biosynthesis of prostaglandins and, subsequently, the protection of the mucosal barrier [158]. These findings are promising, and KLS-GABA is currently in Phase II clinical evaluation for the treatment of chronic low-back pain.

**Table 2:** Preclinical and clinical studies of co-crystals in the context of acute or chronic pain.

Drug Combinations	Doses	Pre-Clinical Study	Clinical Study	Key Findings: Analgesia	Key Findings: Physicochemical Properties	Reference
**Tramadol hydrochloride + Celecoxib co-crystal**	Rat: 0.625–320 mg/kg (i.p. or p.o.)	Postoperative pain model in rats	Moderate to severe acute pain following Bunionectomy + Osteotomy (NCT03108482) or abdominal hysterectomy (NCT3062644)	Rat: Tramadol hydrochloride and celecoxib co-crystal exhibited synergistic analgesic efficacy greater than monotherapy or the theoretical additive effects of the single agents; effects of the co-crystal were comparable to strong opioids, with improved tolerability; reduced ulcerogenic activity	Faster intrinsic dissolution rate of celecoxib and slower intrinsic dissolution rate of tramadol	[146,149,151,152]
	Human: 200 mg twice daily (NCT03108482)			Human (NCT03108482): superior analgesic efficacy of combination twice daily compared with tramadol 50 mg four times daily or celecoxib 100 mg twice daily		NCT03108482
	100 mg twice daily (NCT3062644)			Human (NCT3062644): non-inferior analgesic efficacy to tramadol 100 mg four times daily, improved risk/benefit ratio vs. tramadol alone with lower cumulative opioid exposure		NCT03062644; EudraCT: 2016-000593-38
**Ketoprofen lysine salt (KLS) + Gabapentin (GABA) co-crystal**	Rat: 67.5 mg/kg, 2 cps (acute treatment)	Carrageenan paw edema model in rat	NA	KLS and gabapentin had supra-additive effects in reducing mechanical allodynia and thermal hyperalgesia in NP rats; co-crystal showed lower gastric mucosal damage relative to single compounds	Increased gastric solubility of KLS, consistent with a supersaturation profile	[155]
	Rat: 11.60 mg/kg, 1 cps (repeated treatment for 7 days)	CCI in rat				

Abbreviations: CCI, chronic constriction injury; cps, capsules; i.p., intraperitoneal; KLS, ketoprofen-lysine; p.o., oral; NA, not applicable.

## 6. Conclusions and Future Directions

The heterogenous and multimodal nature of chronic NP makes it a very challenging condition to treat. The underlying etiology of a given peripheral or central NP condition is important to consider in selecting optimal treatment regimens or assessing new data regarding novel therapeutic approaches like those described here. Currently available analgesics are associated with significant safety concerns, including renal toxicity, gastrointestinal toxicity, cardiotoxicity, nephrotoxicity, and abuse liability, underscoring the need to fine-tune the balance of the risk/benefit ratio and closely monitor the tolerability of doses and durations of current treatment prescribed. Multimodal therapeutic approaches represent an important step forward in improving patient outcomes. Drug-drug co-crystallization has the potential to offer several key advantages over combination therapy regimens in this context, improving physicochemical and pharmacokinetic properties of the existing molecules and conveying consequent higher efficacy and/or reduced dose requirements superior to those achieved with combination therapy of the single agents. Critically, the selection of APIs with pharmacodynamic profiles suitable for synergistic efficacy and physicochemical properties which align with stoichiometric constraints is challenging [159]. There are limited examples of drug-drug co-crystals in the pain area that have been published thus far [145]. However, efforts to conduct further studies on co-crystals as a potential solution to chronic NP management are warranted as co-crystallization represents a potential excellent tool to overcome pain management barriers and help achieve optimal efficacy and safety for patients with NP syndromes.

## Figures and Tables

**Table 1:** Preclinical and clinical studies of combination therapies for the treatment of chronic NP.

Drug Combinations	Dose (Human Studies Only)	Pre-Clinical Study	Clinical Study	Key Findings	Reference
**NSAID and Opioids (Including Tapentadol and Tramadol)**					
**Dexketoprofen + Tramadol**	Dexketoprofen, 25 mg Tramadol, 75 mg	Postoperative pain model (plantar incision) in mouse (von Frey test)	Moderate or severe pain following third molar extraction, total hip arthroplasty, or abdominal hysterectomy	Mouse: Additive anti-hyperalgesic effect of combination; inhibition of microglia activation in the spinal cord	[90]
				Human: Combination therapy was superior to either agent as monotherapy; greater peak pain relief of combination over monotherapy, particularly at times >6 h; adverse events were unremarkable	[91,92,93]
**Dexketoprofen + Tramadol**		Acetic acid writhing test, tail-flick test, and formalin test in mouse	NA	Synergistic analgesia, increased risk for constipation	[94]
**Dexketoprofen + Tramadol**		Musculoskeletal pain and complete Freund’s adjuvant inflammatory pain models in mouse (hot plate test, acetone test, and spontaneous pain behaviors)	NA	Synergistic analgesia	[95]
**Ketorolac + Tramadol**		SNI in rat (von Frey test, acetone test, spontaneous pain behaviors)	NA	Synergistic analgesia with subeffective doses of tramadol + ketorolac	[96]
**NSAIDs and gabapentinoids/antiepileptics**					
**Pregabalin + Ketorolac**		SNI in rat (von Frey test, acetone test, spontaneous pain behaviors)	NA	Synergistic analgesia with subeffective doses pregabalin + ketorolac	[96]
**Gabapentin + Naproxen** **Pregabalin + Naproxen**		Carrageenan paw edema model in rat (radiant heat paw-withdrawal test)	NA	Synergistic anti-hyperalgesia with 50:1, 10:1, and 1:1 combinations for assessments of thermal hyperalgesia; only additive effects for paw edema and for 1:50 for thermal hyperalgesia	[97]
**Gabapentin + Ibuprofen**		Formalin test in rat	NA	Additive analgesia	[98]
**Ibuprofen + Oxcarbazepine**		Carrageenan paw edema model in rat (modified paw pressure test)	NA	Synergistic analgesia	[99]
**Gabapentin + Diclofenac**		Postoperative pain model (hindpaw incision) in rat (von Frey test)	NA	Synergistic analgesia with subeffective doses of gabapentin + diclofenac ^1^	[100]
**Gabapentin + Diclofenac**		Formalin test in rat	NA	Synergistic analgesia	[101]
**Gabapentin + Meloxicam**		Chronic constriction injury (sciatic nerve) in rat (von Frey test and acetone test)	NA	Additive anti-hyperalgesia in the von Frey test, synergistic anti-allodynia with the acetone test only at one specific dose tested (10 mg/kg gabapentin + 1 mg/kg meloxicam)	[102]
**Pregabalin + Celecoxib**		Spinal nerve ligation model in rat (von Frey test)	NA	Additive anti-allodynic effect	[103]
**Gabapentinoids and opioids (including tapentadol and tramadol)**					
**Pregabalin + Oxycodone**	Doses titrated to achieve optimal efficacy and tolerability (mean doses at end of study: 141.5 mg + 35.8 mg) (oral)	NA	Moderate to severe neuropathic pain (including failed back surgery syndrome, stenosis medullary spinal canal, post-herpetic neuralgia, painful diabetic neuropathy)	Both combination therapy and oxycodone monotherapy alleviated neuropathic pain; combination therapy was superior to pregabalin monotherapy and allowed a reduction of the dose for both oxycodone (22%) and pregabalin (51%)	[67]
**Pregabalin + Oxycodone**	Pregabalin titrated to achieve optimal efficacy and tolerability (mean pregabalin dose: 227.6 mg/day); oxycodone: 10 mg/day (oral)	NA	Post-herpetic neuralgia or painful diabetic neuropathy	No added benefit of combination therapy	[84]
**Gabapentin + Oxycodone**	Doses titrated to achieve optimal efficacy and tolerability (Gabapentin: 48% of patients: <1200 mg 36% of patients: 12–1800 mg 16% of patients: >1800 mg + Oxycodone: Up to 80 mg daily) (oral)	NA	DPNP	Oxycodone + gabapentin significantly improved pain relief vs. gabapentin alone. Oxycodone + gabapentin co-administration was associated with less escape medication use and fewer nights of disturbed sleep	[78]
**Pregabalin + Tramadol**		L5 spinal nerve ligation model in rat (von Frey test)	NA	Synergistic anti-allodynic effect of combinations as compared to single compounds	[103]
**Gabapentin + Morphine, tramadol, or fentanyl**	Doses titrated to achieve optimal efficacy and tolerability (mean doses at end of study: Gabapentin: 1287.1 mg/day Morphine: 90 mg/day Tramadol: 400 mg/day Fentanyl: 68.1 µg/48 h) (oral)	NA	Nonresponsive neuropathic cancer pain	Stronger reduction in burning and shooting pain after 4 and 13 days and a stronger reduction in allodynia at 4 days with combination therapy relative to opioids alone. Combination therapy group also had significantly fewer side effects	[104]
**Gabapentin + Morphine**		CCI in rat (von Frey and acetone tests)	NA	Synergistic anti-allodynic effects, synergistic anti-hyperalgesia effects; anti-allodynic effect of combination therapy persisted longer than morphine alone 120 m	[105]
**Pregabalin + Morphine**	Doses titrated to achieve optimal efficacy and tolerability (mean doses at end of study: 142.5 mg + 41.8 mg daily) (oral)	NA	Moderate to severe chronic neuropathic pain (including failed back surgery syndrome, stenosis medullary spinal canal, post-herpetic neuralgia, painful diabetic neuropathy)	Combination therapy was more effective in reducing pain intensity at 3 months than pregabalin monotherapy. Furthermore, while reducing the average dosage, combination therapy improved quality of life compared with the patients in the other two groups	[106]
**Gabapentinoids and antidepressants**					
**Pregabalin + Duloxetine** **Pregabalin + Venlafaxine**		Spinal nerve ligation model in rat (von Frey test)	NA	Additive anti-allodynic effect of pregabalin + duloxetine, potentially antagonistic effects with pregabalin + venlafaxine	[103]
**Gabapentin + Venlafaxine**	Doses titrated to achieve optimal efficacy and tolerability (dose range: Gabapentin: 300–3600 mg daily Venlafaxine: 37.5–150 mg daily) (oral)	NA	Painful diabetic neuropathy	Significant improvement in pain reduction, mood, and quality of life observed with combination therapy relative to gabapentin alone	[77]
**Gabapentin + Nortriptyline**	Doses titrated to achieve optimal efficacy and tolerability	NA	Diabetic neuropathy or postherpetic neuralgia	Stronger pain reduction observed with combination therapy relative to either drug as monotherapy	[107]
**Pregabalin + Amitriptyline** **Pregabalin + Duloxetine**	Doses titrated to achieve optimal efficacy and tolerability (mean doses at pain assessment: Amitriptyline + pregabalin: 56 mg + 347 mg Pregabalin + Amitriptyline: 397 mg + 52 mg Duloxetine + pregabalin: 76 mg + 405 mg) (oral)	NA	Moderate–severe DPNP	Combination therapy and monotherapy had similar analgesic effects, but combination therapy had a stronger analgesic effect in those unresponsive to monotherapy	[108]
**Pregabalin + Duloxetine**	300 + 60 mg daily (oral)	NA	Patients with moderate-severe DPNP who were unresponsive to monotherapy	There was no significant benefit observed with combination therapy relative to high-dose monotherapy of either drug on the Brief Pain Inventory Modified Short Form average pain score. The combination was safe and well tolerated	[109]
**Pregabalin + Amitriptyline**	75 + 10 mg daily (oral)	NA	NP	Combination therapy with low-dose pregabalin and amitriptyline was equally effective but more tolerable compared to higher dosage monotherapy with either drug in reducing NP symptom inventory score	[110].
**Additional combinations**					
**Metformin + Ibuprofen** **Metformin + Aspirin** **Metformin + Tramadol** **Metformin + Pregabalin**		Carrageenan paw edema model in rat (von Frey test)	NA	Synergistic analgesia with a ~5-fold reduction of doses of both drugs required for pain relief in all tested combinations	[111]
**Diclofenac + Pyrilamine**		Formalin model in rat Carrageenan paw edema model in rat	NA	Synergistic anti-inflammatory and analgesic effects; Level of gastric damage was reduced with the combination therapy compared with diclofenac alone	[112]
**Diclofenac + Matricaria chamomilla extract (MCE)** **Indomethacin + Matricaria chamomilla extract (MCE)**		Carrageenan paw edema model in rat	NA	Synergistic anti-inflammatory effect (reduced paw inflammation); reduced levels of gastric damage caused by NSAID-MCE combinations compared with monotherapies	[113]
**Diclofenac + α-bisabolol**		Carrageenan paw edema model and formalin test in rat	NA	Synergistic analgesia and ant-inflammatory efficacy (reduced paw inflammation); reduced levels of gastric damage observed with combinations relative to monotherapies	[114]
**Diclofenac + Docosahexaenoic acid (DHA)**		Formalin model in rat Carrageenan paw edema model in rat	NA	Synergistic analgesia and ant-inflammatory efficacy (reduced paw inflammation); reduced levels of gastric damage observed with combinations relative to monotherapies	[115]
**Rofecoxib + Aminoguanidine hydrochloride** **Meloxicam + Aminoguanidine hydrochloride**		CCI model of neuropathic pain in rat (pressure test, hot plate test, cold stimuli paw withdrawal test)	NA	Improved analgesia outcomes (i.e., higher withdrawal thresholds than monotherapies)	[116]
**Gabapentin + Metamizole**		Formalin model in rat	NA	Synergistic analgesia (systemic administration resulted in the highest synergism) ^1^	[117]
**Paracetamol + Oxcarbazepine**		Carrageenan paw edema model in rat and acetic acid-induced writhing test in mouse	NA	Synergistic anti-hyperalgesia	[118]
**Gabapentin + Pregabalin**		Partial sciatic nerve injury (von Frey test)	NA	Subeffective doses of combination potentiated the effects of spinal cord stimulation in neuropathic rats on tactile allodynia and neuronal spinal hyperexcitability ^1^	[119]
**Ibuprofen + Anandamide**		Formalin model in rat Partial sciatic nerve ligation in rat (von Frey test and noxious heat paw withdrawal)	NA	Synergistic analgesia in the formalin model; improved anti-hyperalgesic and anti-allodynic effects in the partial sciatic nerve ligation model (no conclusions regarding additive or synergistic effects)	[120,121]
**Rofecoxib + Anandamide**		Partial sciatic nerve ligation in rat (von Frey test and noxious heat paw withdrawal)	NA	Improved anti-hyperalgesic and anti-allodynic effects in the partial sciatic nerve ligation model (no conclusions regarding additive or synergistic effects)	[120]
**Ketorolac + WIN 55,212-2 (synthetic cannabinoid)**		Acetic acid-induced writhing test and tail-flick test in rat	NA	Additive analgesia in the acetic acid induced writhing test, no effect or added benefit of ketorolac in addition to WIN 55,212-2 in the thermal tail-flick test	[122]
**Ibuprofen + WIN 55,212-2 (synthetic cannabinoid)**		Formalin model in rat	NA	Synergistic analgesia	[123]
**Morphine + WIN 55,212-2 (synthetic cannabinoid)**		CCI (von Frey test and cold allodynia)	NA	Synergistic analgesia (no synergistic effect observed on motor coordination)	[124]
**PhAR-DBH-Me + Tramadol**		Spinal nerve ligation, cisplatin-induced NP (von Frey test)	NA	Synergistic analgesia in rats exposed to spinal nerve ligation but not in rats exposed to cisplatin-induced NP	[125]
**JWH015 (CB2 agonist) + Morphine**		Post-operative pain (plantar incision), SNI, formalin model (von Frey test and thermal withdrawal)	NA	Synergistic efficacy observed in inflammatory, post-operative, and SNI pain; gastrointestinal impairment and conditioned place preference associated with morphine was reduced with combination JWH015	[126]
**Cannabidiol + Tramadol**		STZ-induced diabetic neuropathy (von Frey test)	NA	Additive, but not synergistic, analgesia	[127]
**Morphine + Clonidine**	Doses titrated to achieve optimal efficacy and tolerability (rescue analgesia with paracetamol or dextromoramide was permitted) (i.t.) ^1^	NA	Patients with neuropathic pain after spinal cord injury unresponsive to other treatments	Synergistic efficacy was observed in that morphine + clonidine was associated with stronger pain relief than either as monotherapy or placebo	[128]
**Gabapentin + Ketamine**	300 mg gabapentin 3× daily (oral) + 80 mg ketamine (i.v.)	NA	Patients with neuropathic pain after spinal cord injury	Gabapentin + ketamine produced greater pain relief than did gabapentin alone, but pain returned two weeks after cessation of the ketamine infusion	[129]

Abbreviations: CCI, chronic constriction injury; DHA, Docosahexaenoic acid; DNIC, diffuse noxious inhibitory controls; DPNP, diabetic peripheral neuropathy; i.p., intraperitoneal; i.t., intrathecal; i.v., intravenous; MCE, Matricaria chamomilla extract; NA, not applicable; NSAID, nonsteroidal anti-inflammatory drug; NP, neuropathic pain; OA, osteoarthritis; p.o., oral; s.c., subcutaneous; SNI, spared nerve injury. ^1^ This study assessed the effect of intrathecal drug administration.

## Data Availability

Not applicable.
